# *Alu *repeats increase local recombination rates

**DOI:** 10.1186/1471-2164-10-530

**Published:** 2009-11-16

**Authors:** David J Witherspoon, W Scott Watkins, Yuhua Zhang, Jinchuan Xing, Whitney L Tolpinrud, Dale J Hedges, Mark A Batzer, Lynn B Jorde

**Affiliations:** 1 Dept. of Human Genetics, University of Utah Health Sciences Center, Salt Lake City, Utah, 84112, USA; 2 Yale School of Medicine, New Haven, Connecticut, 06510, USA; 3 Miami Institute for Human Genomics, Miller School of Medicine, University of Miami, Miami, 33124, USA; 4 Dept. of Biological Sciences, Louisiana State University, Baton Rouge, Louisiana, 70803, USA

## Abstract

**Background:**

Recombination rates vary widely across the human genome, but little of that variation is correlated with known DNA sequence features. The genome contains more than one million *Alu *mobile element insertions, and these insertions have been implicated in non-homologous recombination, modulation of DNA methylation, and transcriptional regulation. If individual *Alu *insertions have even modest effects on local recombination rates, they could collectively have a significant impact on the pattern of linkage disequilibrium in the human genome and on the evolution of the *Alu *family itself.

**Results:**

We carried out sequencing, SNP identification, and SNP genotyping around 19 *AluY *insertion loci in 347 individuals sampled from diverse populations, then used the SNP genotypes to estimate local recombination rates around the *AluY *loci. The loci and SNPs were chosen so as to minimize other factors (such as SNP ascertainment bias and SNP density) that could influence recombination rate estimates. We detected a significant increase in recombination rate within ~2 kb of the *AluY *insertions in our African population sample. To test this observation against a larger set of *AluY *insertions, we applied our locus- and SNP-selection design and analyses to the HapMap Phase II data. In that data set, we observed a significantly increased recombination rate near *AluY *insertions in both the CEU and YRI populations.

**Conclusion:**

We show that the presence of a fixed *AluY *insertion is significantly predictive of an elevated local recombination rate within 2 kb of the insertion, independent of other known predictors. The magnitude of this effect, approximately a 6% increase, is comparable to the effects of some recombinogenic DNA sequence motifs identified via their association with recombination hot spots.

## Background

Approximately one-half of the human genome consists of the remnants of past transpositional bursts [1]. *LINE-1 *non-LTR retrotransposons and the *Alu *elements they mobilize continue to replicate in the human gene pool to this day [2]. As a result of *Alu *retroposition, our genomes are littered with more than one million small (~300 bp), non-allelic regions whose DNA sequences are nearly identical to each other. Their recombinogenic impact is evident: these scattered homologies trigger non-allelic homologous recombination (NAHR) events that lead to translocations, deletions, duplications, and other chromosomal abnormalities and copy number variations [2-6]. These events have affected the long-term evolution of the human genome and of the *Alu *insertions themselves [7-11]. *Alu *repeats have been implicated in differential methylation states of the genome, in the translation response to cellular stress, and in the regulation of transcription [2]. However, the impact of *Alu *insertions on the rates of allelic recombination events in the human germline remains largely unknown. It has been suggested that polymorphic *Alu *insertions may suppress recombination when found in the heterozygous state [12], and fixed *Alu *insertions may contain specific DNA sequence features capable of recruiting recombination-enhancing or -suppressing factors.

Meiotic recombination rates in humans vary widely across the genome [13]. The search for the causes of this variation initially focused on broad-scale DNA sequence and chromosome-level features, such as G+C and CpG content, or the density of poly(A)/poly(T) stretches and protein-coding genes [14,15]. Although these features explain nearly half of the variance in recombination rate at the 5 Mb scale, they explain less than 5% of the variance of recombination at the 5 kb scale [16]. More recently, attention has turned to DNA sequence motifs associated with recombination "hot spots," where many recombination events are concentrated [16-20]. A family of short (~7-13 bp) hot spot-associated motifs may account for a sizable proportion of those hot spots and thus for a substantial proportion of the variance in recombination rate. These motifs are common outside of *Alu *elements and in other repeat sequences (e.g. THE1A/B elements), but some *Alu *elements carry those motifs [20]. That association translates into a slight enrichment of several *Alu *subfamilies in hot spots (e.g., 1.1-fold for *AluY*), and consequently an association with higher recombination rates [20]. However, that effect appears to be due entirely to the recombinogenic motifs: to the extent tested, no association was found between *Alu *insertions lacking the motifs and higher recombination rates [20]. These negative results imply that the *Alu *sequence is not uniquely nor highly recombinogenic in itself.

Since previous studies have analyzed recombination rate variation at a broad scale, or have focused mainly on hot spots, a less dramatic effect (not rising to the level that would be detected as a hot spot), or an effect mediated only by a minority of more recently-inserted copies, would have gone undetected. Yet even if the impact of individual *Alu *insertions on local recombination rates is small, the sum of those effects over the very large number of *Alu *insertions in the human gene pool could have a significant cumulative impact on the structure of our genomes. Moreover, any effect of *Alu *insertions on recombination rate in their immediate vicinity could influence their own evolutionary fates, the evolution of the *Alu *retroposon family, and the evolutionary responses of the genetic pathways that regulate recombination itself.

Here we focus specifically on the effect of recent (less than 10% diverged from consensus) *AluY *insertions. Of all the repeat families in the human genome, the *AluY *subfamily has the largest number of recently inserted copies. Any *Alu*-specific properties that affect recombination should be most apparent in young insertions, rather than older insertions that have accumulated many mutations that may have altered their properties. The high copy number of *AluY *insertions provides the statistical power needed to detect modest effects, and the homogeneity of the subfamily reduces the danger of missing an effect due to heterogeneity within the data set. Our question is: does the presence of an *AluY *insertion affect the local rate of recombination? We show that the presence of a fixed, young *AluY *insertion is significantly predictive of a modestly elevated local recombination rate.

## Results

In order to address the effect of *Alu *insertions on local recombination rates as directly and clearly as possible, we sought to eliminate or account for factors and biases that could affect recombination rate estimates. In short, we first constructed data sets that avoid complicating factors and biases and then used covariates in stepwise linear regression analyses to account for the remaining factors. The basic unit in our analyses is a ~50 kb region containing a single *AluY *insertion locus and common SNPs spaced at 4-5 kb intervals throughout each region. The exact size of any particular "*AluY *region" is determined by the locations of the first and last SNP ascertained for that region. By focusing on regions with just one *AluY *insertion, we avoid modeling complex interactions between multiple *AluY *insertions in one or several inter-SNP intervals. By maintaining uniformity of inter-SNP interval sizes, we avoid biases in the estimation of recombination rates on intervals of very different sizes. The frequency of common SNPs in the human population and our need for uniformly-sized intervals across many *AluY *regions constrain our choice of SNP spacing intervals. Under those constraints, the 4-5 kb SNP spacing best meets our goal of estimating recombination rates in small intervals. We used this same strategy to select *AluY *regions and uniformly-spaced SNPs from our own "world diversity panel" (below) and from the HapMap Phase II data.

**Figure 1:**
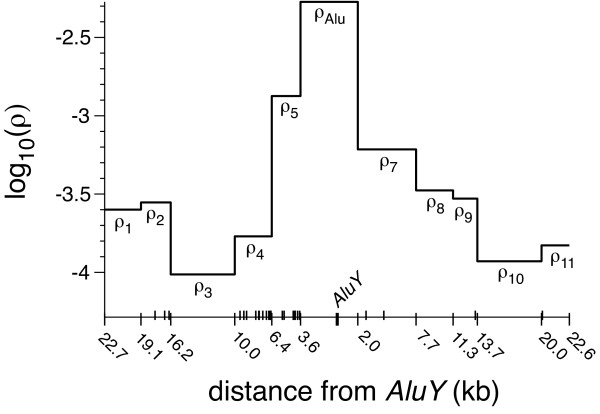
**A typical genomic region surrounding a focal *AluY *element**. Estimates of the recombination rate parameter *ρ *(log_10 _scale) are shown for the eleven inter-SNP intervals. The sixth *ρ *-estimate (labeled *ρ*_Alu_) is for the interval containing the *AluY*, which has the highest recombination rate in this particular region. The positions of the 12 SNPs chosen for analysis are shown relative to the center of the *AluY*; other SNPs in the region are indicated by small tick marks. This region spans ~45 kb on chromosome 7, centered on the *AluY *at 32,081,567 bp (UCSC hg18; [35]).

After selecting *AluY *regions and SNPs within them, we used the genotypes at those SNPs in various population samples to estimate the rescaled recombination rate parameter (*ρ*) for each inter-SNP interval. A typical *AluY *region, with *ρ *estimates plotted for each inter-SNP interval, is shown in Figure 1. The values of other covariates for each interval were computed as detailed in the Methods section. Stepwise linear regression was used to ascertain whether the presence of an *AluY *insertion locus in an inter-SNP interval significantly changes the recombination rate in that interval, relative to the rate in intervals that do not contain an *AluY *insert.

### *AluY *regions in world diversity panel

We designed our first data by ascertaining evenly spaced common SNPs from a panel of samples drawn from Africa, Asia, and Europe, then genotyping those SNPs in our population samples from those continental groups (see Methods). Our stepwise linear regression analyses detected a significant positive effect (2.5-fold above the expected value, *p *= 0.033) of the presence of a fixed *AluY *insertion on the local recombination rate in the African subset of our world population diversity sample (Table 1). As expected, both the regional mean recombination rate and the percent G+C in an interval significantly predicted the recombination rate. The *a priori *expected effect of hot spots is slightly weaker and does not reach statistical significance. No significant evidence of an effect of fixed *AluY *insertions on recombination was found in the East Asian or European data subsets. We also found no evidence that the five polymorphic *AluY *insertions influenced local recombination rates in African, East Asian, or European population samples (Table 1). The means and standard deviations of the variables are shown in Table 2. Terminal inter-SNP intervals (those delimited by the terminal and sub-terminal SNPs in each *AluY *region) were excluded from the regression analyses out of concern that their recombination rate estimates might be downwardly biased (see Methods).

**Table 1:** Tests of effects of fixed and polymorphic *AluY *insertions on local recombination rates. *P*-values for the significance of the *AluY *presence variable are italicized.

	*AluY *regions	Total Intervals^b^	Africa	Europe	East Asia
Polymorphic	5	30	*0.82*	*0.69*	*0.95*
Fixed	14	99	*0.033*	*0.71*	*0.35*

# individuals^a^			138 to 147	96 to 108	67 to 74

^a ^Number of individuals used to infer recombination rates, minimum to maximum (number varies between regions because of individuals removed due to missing data).

^b ^Total number of inter-SNP intervals analyzed for a data set.

**Table 2:** Means and standard deviations of the regression variables, by data set.

Data Set	Numbers of Regions (Intervals)	Interval log_10_(*ρ*) (s.d.)	Interval length, bp (s.d.)	Regional log_10_(*ρ*)^a ^(s.d.)	Interval G+C (s.d.)	Core motif count (s.d.)	Ext. motif count (s.d.)	Hot spot (s.d.)	*AluY* (s.d.)	*AluY *length, bp^b^ (s.d.)
Diversity panel: Africa, fixed	14	-3.34	5,240	-3.44	0.383	1.00	0.323	0.131	0.141	291
	(99)	(1.09)	(2,840)	(0.705)	(0.0664)	(1.55)	(0.636)	(0.339)	(0.35)	(48.4)

HapMap YRI, fixed	6,235	-3.60	4,210	-3.43	0.403	1.18	0.358	0.142	0.143	301
	(43,645)	(0.844)	(1,270)	(0.837)	(0.0573)	(1.56)	(0.735)	(0.349)	(0.350)	(13.3)

HapMap CEU, fixed	5,344	-4.13	4,210	-3.96	0.403	1.18	0.357	0.142	0.143	301
	(37,408)	(0.981)	(1,230)	(1.03)	(0.0570)	(1.56)	(0.741)	(0.349)	(0.350)	(13.1)

^a ^For each interval, the regional log_10_(*ρ*) is the weighted average taken over all intervals in the region, excluding that focal interval.

^b ^*AluY *length statistics are given for descriptive purposes. Only the presence or absence of an *AluY *is used as a regression variable.

The statistical power of this data set of 14 fixed *AluY *regions and 5 polymorphic regions is limited to detecting large effects. The significant association between *AluY *insertions and increased recombination observed in the African sample, but not in the non-African samples, likely reflects the earlier founding and larger effective population size of the African population [21]. These attributes increase the number of detectable recombination events, and thus the statistical power to detect factors associated with recombination, in this population.

### Inter-SNP interval length, recombination rate, and *AluY *insertions

To increase the power to detect any association between *AluY *elements and recombination rate, we used data from the HapMap project (phase II). This large data set provides estimates of the inter-SNP recombination rate for every inter-SNP interval in the data [22]. Before making use of this resource, however, we examined the data set for biases that might impede our ability to detect an effect of *Alu *elements on recombination. Our initial analyses of the HapMap data found that, in general: (1) longer-than-average inter-SNP intervals have lower-than-average estimated recombination rates (regardless of whether they contain *AluY *insertions or not); (2) inter-SNP intervals with *AluY *insertions in them are longer than intervals without them; and (3) *AluY *insertions are associated with both longer-than-average intervals and lower-than-average estimated recombination rates. Specifically, among 3,088,316 autosomal inter-SNP intervals with lengths between 10 and 10,000 bp for which recombination rates were estimated by the HapMap project, a linear regression of recombination rate (cM/Mb, log_10_-scaled) on interval length (log_10_) yields a significantly negative slope (-0.161, *R*^2 ^= 0.01, *p *<< 10^-50^). Of those intervals, 107,189 contain at least part of an *AluY *insertion (of the class we selected for analysis; see Methods). Consistent with the overall pattern, those intervals are longer, on average, than intervals without an *AluY *insertion (2,243 bp vs. 739 bp; length distributions differ significantly, two-sample *t*-test, *p *<< 10^-50^). Again consistent with the general pattern, the estimated recombination rate in these longer, *AluY*-containing intervals is lower than in the shorter intervals that lack *AluY *insertions (two-sample *t*-test, *p *<< 10^-50^).

The reason for the larger average size of intervals with an *AluY *in them (or equivalently, the lower density of genotyped SNPs near *AluY *insertions) is unclear. It might be due to the difficulty of designing robust genotyping assays for SNPs near repeat sequences in the context of a high-throughput genotyping project, or perhaps these repeats are in fact associated with lower nearby genetic variation. We observed a similar pattern with *LINE-1 *insertions (not shown). The cause of the general association between estimated recombination rate and inter-SNP interval length in HapMap data may be an artifact of the estimation procedure, since regions of lower SNP density contain less information about past recombination events.

We therefore eliminated the potentially confounding relationship between inter-SNP interval size and estimated recombination rate by selecting uniformly sized intervals (see Methods), re-estimating the recombination rates in those intervals, and also including interval length as a covariate in our regression analyses. Previous analyses of correlations between *AluY *insertions and local recombination rates did not account for this bias and were focused on larger effect sizes, which may explain why no *AluY*-specific effect was detected [20].

### *AluY *Regions in HAPMAP YRI trios

To further test the initial results we observed in the African sample of our world diversity panel, we assembled a genome-wide data set of HapMap SNPs typed on the 30 Yoruba (YRI) parent-child trios [23]. *AluY *insertions were identified by RepeatMasker (UCSC Genome Browser table, http://genome.ucsc.edu/) and excluded known polymorphic insertions by comparison with dbRIP [24]. We selected 6,235 *AluY *regions and uniformly-spaced SNPs within them (defining 43,645 inter-SNP intervals) from the HapMap data using an adaptation of the method we used on our world diversity panel above (see Methods). We analyzed these data after removing the terminal and sub-terminal intervals from each region (four intervals per region) to eliminate edge effects. Table 2 shows the means and standard deviations of log_10_(*ρ*) for these intervals and for the seven predictor variables defined for each inter-SNP interval: length, regional recombination rate, G+C content, "core" motif count, "extended" motif count, hot spot presence, and *AluY *presence (as detailed in Methods).

After accounting for all other effects in the model, we find that the presence of an *AluY *insertion predicts a statistically significant (*p *< 0.00014) though modest (~5%) increase in recombination rate. The length of an interval has a significant but very small effect on the recombination rate in that interval, which implies that our strategy to eliminate the interval length factor was successful. Each of the other variables is independently predictive of the local recombination rate. The impacts of the regional recombination rate and G+C composition are the largest and most significant, as expected. The hot spot-associated recombinogenic motifs are associated with small, local variations in recombination rate independently of their association with hot spots. Since those motifs are present in a minority of hot spots [20], it is not surprising that hot spots themselves have an independent and much stronger effect: an interval that overlaps a known hot spot has a 2.3-fold greater recombination rate, on average, compared with nearby intervals that do not overlap a hot spot.

### *AluY *regions in HAPMAP CEU trios

With our initial set of 14 *AluY *regions, we detected no effect of fixed *AluY *insertions on recombination in our European population sample. We then asked the same question using 5,344 *AluY *regions (around fixed *AluY *insertions, containing 37,408 inter-SNP intervals, exclusive of terminal and sub-terminal intervals) and 30 CEU parent-child trios genotyped by the HapMap project. Means and standard deviations of the regression variables are shown in Table 2.

Regression analysis (Table 3) shows that the presence of a fixed *AluY *insertion is associated with an ~8% increase in the recombination rate in an interval (*p *< 1.7 × 10^-7^). The overall results are very similar to those observed with the YRI data. This similarity is expected, since the patterns of variation and linkage disequilibrium in the CEU population sample are correlated with those in the YRI sample because of their shared ancestry. The effect of interval length on recombination rate is small, as it was with the YRI data, and statistically insignificant in this case.

**Table 3:** Stepwise linear regression results (effect size coefficient, standard error, and *p*-value) for each variable, by data set. *P*-values < 10^-50 ^are shown as 0.

Data Set		Interval length	Regional log_10_(*r*)	Interval G+C	Core motif	Extended motif	Hot spot	*AluY *presence
Diversity panel: Africa, fixed	Coefficient	-1.07 × 10^-7^	1.29	-2.69	-0.0439	-0.0725	0.250	0.395
	Std. Err.	2.27 × 10^-5^	0.0919	0.978	0.0572	0.104	0.202	0.182
	*p*-value	0.996	7.3 × 10^-25^	0.0071	0.44	0.49	0.22	0.033

HapMap YRI, fixed	Coefficient	-6.17 × 10^-6^	0.824	0.625	0.00891	0.0296	0.356	0.0221
	Std. Err.	1.66 × 10^-6^	0.00248	0.0408	0.00146	0.00283	0.00570	0.00579
	*p*-value	2.0 × 10^-4^	0	0	9.4 × 10^-10^	0	0	1.4 × 10^-4^

HapMap CEU, fixed	Coefficient	2.90 × 10^-6^	0.815	0.555	0.00837	0.0378	0.389	0.0320
	Std. Err.	1.86 × 10^-6^	0.00221	0.0443	0.00154	0.00304	0.00624	0.00611
	*p*-value	0.12	0	0	6.1 × 10^-8^	0	0	1.7 × 10^-7^

Approximately 16% (842) of the *AluY*-containing intervals in the CEU data set also overlap a hot spot. While that does not mean that those hot spots overlap the *AluY *insertions themselves, we nonetheless checked for a potential interaction effect between hot spots and *AluY *insertions, since such an interaction could account for part of the *AluY *effect. We observed a significant interaction effect in the CEU HapMap data set (using a 0/1 indicator for the interaction, *p *< 0.006, effect coefficient ± standard error: 0.047 ± 0.0057). Independent effect sizes for *AluY *and hot spots were slightly but not significantly reduced (coefficient ± standard error: 0.0248 ± 0.0066 and 0.381 ± 0.0068, respectively; compare to Table 3). Both effects remain clearly significant, and the results for other factors are only trivially affected. No significant hot spot × *AluY *effects were seen in the other data sets.

The differences between the results based on our world diversity panel and those obtained from the HapMap data should be considered with caution, due to the small number of regions genotyped in the former. An effect size of ~8% would not be detectable with a sample size of 99 intervals in 14 *AluY *regions, so the negative results obtained in our European and East Asian samples are probably due to a lack of statistical power. Although the 95% confidence intervals around the effect sizes estimated for the fixed *AluY *insertions in the sub-Saharan African and the HapMap YRI data sets do not overlap, the 99% confidence intervals do (see standard errors in Table 3).

**Figure 2:**
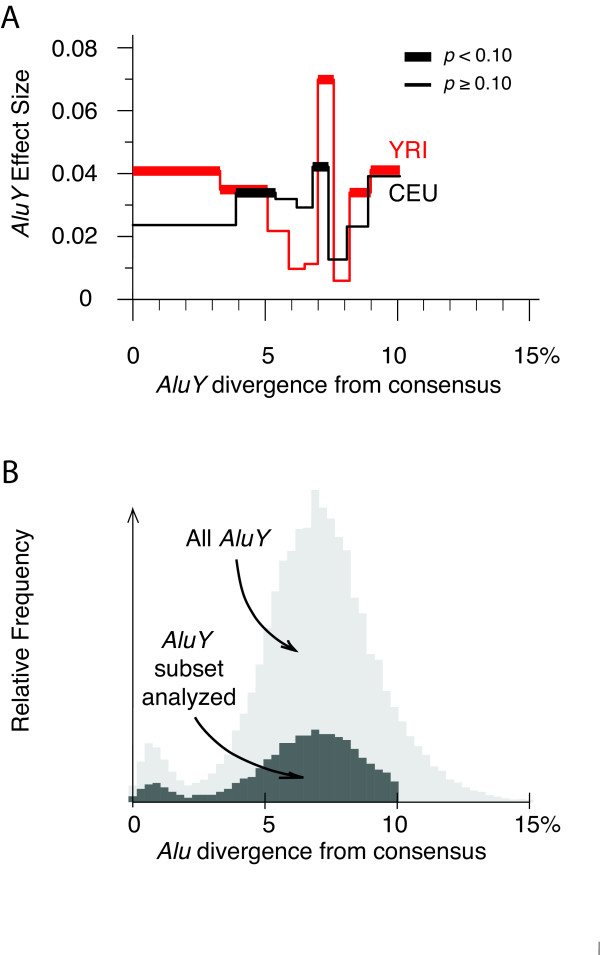
**Size and significance of the effect of *AluY *insertions on the local recombination rate**. (A) The effect of *AluY *insertions (linear regression coefficient for the *AluY *variable) is plotted against the percent divergence of *AluY *elements (binned into non-overlapping groups of approximately uniform number, i.e., 600-800 elements; divergences taken from RepeatMasker). The red and black lines correspond to results from the HapMap YRI and CEU data sets, respectively. (B) Histogram of *AluY *element frequencies vs. percent divergence from their respective subfamily consensus sequences. Only elements between 250 and 350 bp long, with no more than 10% of their sequence deleted or composed of non-*Alu *insertions, were counted. The dark gray histogram shows the distribution of *AluY *elements that were chosen for regions analyzed in this work (magnified vertically by fivefold for visibility), while the light gray histogram includes all *AluY *elements. The horizontal axes in both panels are identically scaled and aligned.

The majority of *AluY *insertions are 10-40 million years old (since 1% divergence ≈ 4 MYr for human *Alu *insertions; [2,25]). The accumulation of mutations in *AluY *copies as they age might alter their effects on the local recombination rate. Figure 2(A) explores how the effects of *AluY *insertions on recombination depend on their degree of sequence divergence from their subfamily consensus sequences, and Figure 2(B) shows the relative frequencies of *AluY *insertions plotted against their divergence. A similar recombination rate effect is seen across all classes of the *AluY *divergence spectrum.

## Discussion

We have assessed whether *AluY *insertions affect the recombination rate in their immediate neighborhood. We first generated and analyzed a data set of *AluY *insertions and surrounding SNPs that were ascertained to limit extraneous factors and thus to maximize our ability to detect such effects. To test the observations gained from those data, we extended the ascertainment design and analyses to a larger set of *AluY *insertions and neighboring SNPs extracted from the HapMap Phase II data. Because *AluY *insertions are correlated with some sequence features (e.g. high G+C content, recombinogenic motifs) that are themselves associated with higher recombination rates or with recombination hot spots, we included those features as covariates in our analyses. We included hot spots themselves as proxies for the as-yet-unknown factors that presumably cause those hot spots.

As expected, the average recombination rate within ~15 kb on either side of an *AluY*-containing interval was a strong predictor of that interval's recombination rate. While this yields no insight about the cause of that broad-scale variation, it allows us to factor out any effects at that scale. Even with the mean surrounding regional recombination rate already factored out, the G+C content of an inter-SNP interval is strongly predictive of its recombination rate. The G+C content itself is correlated with the "core" and "extended" hot spot-associated recombinogenic motifs, since they are GC-rich. Nonetheless, both of those motifs carry additional significant predictive power. As expected, the presence of a hot spot in (or overlapping) an interval has a much stronger effect, increasing the recombination rate by ~2.4-fold, on average. There is a slight association between hot spots and *AluY *insertions (consistent with [20]): inter-SNP intervals that contain an *AluY *are 13% more likely to overlap a hot spot than control intervals are (in both HapMap YRI and CEU data; *p *< 0.001, binomial tests). Some degree of association would be expected under the hypothesis that *AluY *insertions increase the local recombination rate, since they would push that rate past the threshold for hot spots in at least some regions. There is also some evidence for a positive interaction between hot spots and *AluY *insertions (albeit only in the CEU data set; see Results). However, since many unknown factors may interact to generate recombination hot spots, and since an *AluY*-specific effect should be detectable independently from those factors and the hot spots they generate, we have attempted to factor out the effect of hot spots.

After factoring out effects that are not specific to *AluY *sequences, we still find that the presence of a fixed *AluY *insertion has a significant positive impact on the recombination rate within the ~4 kb inter-SNP interval that contains it. A fixed *AluY *insertion appears to cause a twofold enhancement of the local recombination rate in the 14 *AluY *regions we genotyped in our sub-Saharan African sample. A smaller positive effect - a 6.4% increase over the surrounding intervals, on average - is strongly evident in the larger HapMap-based data sets, for both the YRI and CEU populations.

No relationship between polymorphic *AluY *insertions and the local recombination rate was found in the five regions genotyped in our world diversity panel, but a modest effect (as observed for fixed *AluY *insertions) would not be detectable in a data set of that size. We therefore turned to the HapMap YRI trio data set to test for a smaller effect of polymorphic *AluY *insertions on the local recombination rate. Using the methods we applied above to ascertain fixed *AluY *regions, we identified 552 polymorphic *AluY *regions based on the *AluY *loci in dbRIP [24]. We examined 3,864 inter-SNP intervals (terminal intervals excluded to eliminate edge effects) and found no significant effect of the presence of polymorphic *AluY *elements on local recombination rates.

The magnitude of the per-copy effect of a fixed *AluY *on the local recombination rate is comparable to the effect of the stronger of the two recombinogenic motifs that we analyzed (Table 3). Given the resolution of our data sets (~4 kb SNP spacing), it is possible that the effect may be stronger but more localized than we have reported, since the effect is diluted out over the entire *AluY*-containing interval. In considering potential causes of the observed effect, it must be noted that the recombination rates estimated here reflect only the history captured by human SNPs, nearly all of which arose less than 1.5 MYr ago. Thus *AluY *characteristics that existed only prior to that time (e.g. the past polymorphic status of now-fixed *AluY *insertions) cannot explain the recent effect of those insertions.

*AluY *sequences might bind cofactors or influence chromatin structure in a way that influences the local recombination rate, as has been suggested for some short recombinogenic motifs [20]. For example, *Alu *insertions are typically flanked on both sides by target sites for *LINE-1 *endonuclease. This is because *Alu *insertions are created by *LINE-1*-encoded proteins [26] at *LINE-1 *endonuclease cutting sites, and the original target sites are duplicated during the insertion event. *Alu *insertions may thus attract *LINE-1 *endonuclease, which creates double-strand breaks (DSBs) in the DNA that can then be resolved as recombination events. *LINE-1 *endonuclease generates large numbers of DSBs [27], which suggests that endogenous *LINE-1 *activity might generate DSBs at a rate sufficient to affect recombination rates.

## Conclusion

In summary, we have demonstrated that the presence of a fixed *AluY *element enhances the local recombination rate by approximately 6%. This effect is similar in magnitude to that observed for previously identified recombinogenic motifs. While the effect of each *AluY *element is relatively small, the presence of hundreds of thousands of these elements throughout the human genome implies that they exert a substantial effect on genome-wide recombination rates. Further research is needed to identify precisely the molecular mechanism responsible for this effect.

## Methods

### World diversity panel SNP ascertainment and genotyping

We first identified genomic regions of ~50 kb in size, each centered around a single *AluY *insertion, with no other young *AluY *insertions or known genes in the region. Nearly all of these less-diverged elements are of the *AluY *subfamily, so we restrict our analysis to *AluY *elements. We selected 19 such *AluY*-containing regions based on previous characterization [28,29]. Fourteen of these are defined by a fixed but recently inserted *AluY *element, and five are defined by *AluY *insertions that are still polymorphic for presence or absence in humans. We then identified a total of 206 SNPs in these regions by searching public databases and by resequencing 1 kb stretches of DNA at ~5 kb intervals flanking the *AluY *insertion loci in seven individuals of African, European, and East Asian ancestry (21 total). This ascertainment design delivers evenly spaced SNPs and reduces the ascertainment bias for common European SNPs that pervades most large publicly available data sets, such as the HapMap data. We then genotyped these SNPs in 347 individuals sampled from Sub-Saharan Africa (152), Europe (118), and East Asia (77) (additional population details can be found in [30]).

Sequencing of both strands in the targeted loci on the ascertainment panel of 21 individuals was carried out in our laboratory using an ABI 3100 sequencer (Applied Biosystems) and primer designs based on the human reference genome. SNPs were identified using PolyPhred [31], and those with MAF > 5% in the 21 individuals were selected for genotyping in the 347 individuals in our sample using standard methods (SNaPshot^®^, Applied Biosystems).

### Recombination rate estimation and model variables

For each *AluY *region-by-continent data set, we used the default recombination rate model in PHASE 2.1 (MR0; [32]) with a segment size of 12 markers, 200 burn-in iterations followed by 100 sampling iterations, with a 10-times longer final run (using all loci instead of segments) for better sampling of the recombination rate estimates. We ran the entire estimation process five times and used results from the run with the best average goodness-of-fit. Our estimate of *ρ *for each interval is the median of the final sampled values produced by PHASE for that interval, as recommended by the authors.

Where parent-offspring trios were available, we used them to obtain better haplotype reconstructions and recombination rate estimates. Within trios, genotypes that were incompatible with Mendelian inheritance were treated as missing data. Data subsets (genotypes for all SNPs in an *AluY *region, in all individuals in the continental population sample) that exhibited more than 10 Mendelian inheritance conflicts were not analyzed. On a region-by-region basis, any individual with more than 50% missing data was removed (along with its entire trio, where applicable).

We estimated the rescaled recombination rate parameter, *ρ*, for every inter-SNP interval in each *AluY *region from the genotypes of SNPs in each region using PHASE 2.1 [32]. Recombination rates were estimated separately for each continental group (Africa, Europe, or Asia).

We then used stepwise-fitted linear regression (stepwisefit in Matlab; [33]) to test for an effect of *AluY *insertions on recombination rates while controlling for other factors that might also influence recombination rates. Our regression model predicts *ρ *for each inter-SNP interval (across all *AluY *regions) as a function of seven variables:

(1) The length of the inter-SNP interval (log_10 _scale), which allows us to factor out and test for a potentially confounding relationship between interval length, recombination rate, and *AluY *presence or absence.

(2) The mean *ρ *of all intervals in the *AluY *region other than the interval containing the *AluY *(see Figure 1). This essentially uses the inter-SNP intervals surrounding the *AluY*-containing interval as matched controls (see Figure 1) and will factor out broad-scale variation in the recombination rate, regardless of the cause. In particular, this should account for biases that might have been introduced by the procedure that we used to select *AluY *regions (such as slightly lower *AluY *density or slightly higher SNP heterozygosity compared to genome-wide averages).

(3) The G+C base pair composition of the interval. This is known to be correlated with recombination rates at broad scales [14,15,34] and might well have a short-range effect.

(4) The number of copies per interval of a 7-bp GC-rich "core" motif (CCTCCCT) that is associated with recombination hot spots [16].

(5) The number of copies per interval of an "extended" degenerate motif (CCNCCNTNNCCNC) that is associated with recombination hot spots [20]. The core and extended motifs are sometimes found in *AluY *copies, and their potential recombinogenic effects might explain some or all of any effect that *AluY *insertions might have on the recombination rate. Instances of each motif and its reverse complement were identified and counted in the UCSC hg18 reference human genome sequence.

(6) An indicator variable, indicating whether or not an interval overlaps a hot spot (0 or 1, respectively; hot spot locations as identified in [16]). Although hot spots are not DNA sequence features *per se*, they do correspond to small (mostly <10 kb) regions where recombination rates several times higher than in the surrounding regions. In effect, we use the presence of a hot spot as a proxy for the unknown local genomic features that presumably cause hot spots.

(7) Lastly, the variable of interest in this work: an indicator of the presence (1) or absence (0) of an *AluY *insertion in the interval.

In an alternative test of the independence of the effects of *AluY *insertions and the recombinogenic motifs that sometimes occur in them, we generated subsets of the HapMap data by removing all *AluY *regions where the focal *AluY *contained either recombinogenic motif. Our findings remained essentially unaltered, indicating that the effects of the motifs were well accounted for in the linear regression models.

Although it was possible to identify many ~50 kb regions in the human genome that contained exactly one young *AluY *insertion, it was not possible to find such regions that were also free of copies of other repeat sequence families, because those are too common. If copies of other families affect recombination and are strongly correlated with young *AluY *insertions at the ~4 kb scale we used, those effects could cancel out or be confounded. To test this possibility for *LINE L1*, *HERV*, *Alu *repeats other than *AluY*, and DNA repeats (separately), we constructed additional variables that indicate whether an inter-SNP interval overlaps a repeat of that class or not (1 or 0; similar to the hot spot variable). Those four classes account for the vast majority of non-*AluY *repeats in the genome. Adding these new variables into our linear regression models caused only trivial and statistically non-significant changes in the size of the effect of *AluY *repeats on recombination: the effect size coefficients changed by no more than 4% of the original estimate at most, and by only 0.7% on average. Thus other repeat sequences can be safely treated as uncorrelated background effects.

### "Edge" effects on recombination rate estimation

PHASE relies on the pattern of linkage disequilibrium between SNPs to infer the historical rates of recombination in the genomic intervals defined by those SNPs. For intervals at the end of a region of neighboring SNPs, however, there are no further SNPs on one side. This could limit the ability of PHASE to detect evidence of past recombination events, which could result in a downward bias in recombination rate estimates for those intervals. The *AluY *insertions in our data are nearly always in the central interval of each region, where information about recombination events should be sufficient for unbiased estimates. Lower recombination rate estimates in terminal intervals due to an "edge effect" could cause recombination rates at internal intervals (in particular, the *AluY*-containing intervals) to appear significantly higher than the local average.

We examined our data for edge effects by including additional indicator variables in the linear regression model described above. These variables indicated whether or not (1 or 0) and interval was terminal, sub-terminal, sub-sub-terminal, and so on, in its region. In the HapMap data sets, where each region has eleven intervals, this requires five variables. In the large data sets for fixed *AluY *in the HapMap YRI and CEU samples (comprising 6,235 and 5,344 regions, respectively), terminal intervals had a modestly but significantly lowered recombination rate (effect coefficients -0.018 and -0.025, p < 10^-9 ^and *p *< 10^-4^, for YRI and CEU data sets respectively). Sub-terminal intervals exhibited a similar effect in the YRI data set only (effect size -0.26, *p *< 10^-7^). Other internal intervals (apart from the central *AluY*-containing interval) showed no position effects. The remaining data sets derived from our world diversity panel are all much smaller, and no edge effect was detected for the terminal or sub-terminal intervals there.

In order to eliminate edge effects from our data, we removed the terminal and sub-terminal intervals from all linear regression analyses on the HapMap-derived data sets (for fixed *AluY *in the YRI and CEU samples, and for polymorphic *AluY *in the YRI sample). In the smaller data sets derived from our world diversity panel, the *AluY *are not as tightly correlated with the central interval in a region; in one case, the *AluY *is in a sub-terminal interval. For these data sets, we nonetheless dropped all terminal intervals from all analyses. The exclusion of terminal and/or sub-terminal intervals has some quantitative impacts, but does not qualitatively affect the results of any of our analyses.

### HapMap data set construction

To extend our analysis to a different set of individuals and a larger set of loci, we turned to the publicly available HapMap data set [23]. We analyzed data from the parent-child trios of European (CEPH) and Yoruban ancestry (YRI; thirty trios each; [23]. Since we are interested in the effects of typical *AluY *insertions, we searched the UCSC Genome Browser [35] "rmsk" database table (itself generated using RepeatMasker[36]) for all *AluY *insertions in the human genome reference sequence that met the following criteria: (1) the insertions have been mapped to a specific location in an autosome, (2) they are no more than 10% diverged from their respective *AluY *subfamily consensus sequences, (3) they are between 250 and 350 bp in length, (4) no more than 10% of any insertion consists of inserted non-*AluY *sequence, (5) no more than 10% of the subfamily consensus sequence is missing from the insertion. We identified 113,852 such *AluY *insertions.

We then emulated the *AluY *region and SNP ascertainment strategies that we used for our own genotyping project ("World diversity panel SNP ascertainment and genotyping," above) to select comparable *AluY *regions and evenly-spaced SNPs from the HapMap data. Out of the *AluY *insertions identified as above, we selected only those that had no other *AluY *fragments (or known polymorphic *Alu *insertion loci in dbRIP; [24]) within 25 kb of them, in order to simplify analysis and eliminate potentially complicating interactions between neighboring *AluY *elements. We further winnowed this set down to those *AluY *insertions that had at least twelve SNPs with MAF>0.1 (in the population being analyzed) within 25 kb of the *AluY *locus. We used the more demanding 10% MAF threshold (instead of 5%) in order to obtain better recombination rate estimates. Among SNPs that met those criteria, we chose sets that maximized the uniformity of the inter-SNP interval lengths (as our own ascertainment procedure above did). We then ranked the HapMap *AluY *regions according to the evenness of SNP spacing and selected regions with the highest evenness for further analysis. *AluY *regions and SNPs were ascertained separately for the HapMap YRI and CEU data sets. For the analysis of polymorphic *AluY *insertions, *AluY *regions with 12 SNPs each were selected and constructed as above, after using liftOver [37] to translate dbRIP chromosomal positions from UCSC hg17 to hg18 positions.

The SNPs discovered in this work have been submitted to dbSNP. All the data sets used in this work (*AluY *regions, SNP loci, genotypes, recombination rate estimates and all other predictor variables computed on inter-SNP intervals) are available from the authors on request.

## Authors' contributions

DW participated in the conception of this study, designed and carried out the statistical analyses, and drafted the manuscript. WW participated in the design of the data collection strategy and in the ascertainment and genotyping of variants in our world diversity panel. YZ participated in the sequencing and genotyping. JX participated in designing the study and in preparing the manuscript. WT participated in genotyping of variants in the world diversity panel. DH participated in the ascertainment of *Alu *polymorphisms used in this study. MB participated in the conception, design and coordination of this study. LJ conceived of the study and participated in its design, coordination, and in manuscript preparation. All authors read and approved the final manuscript.
